# A phased strengthening of laboratory capacity in the Eastern Mediterranean Region during the COVID‐19 pandemic

**DOI:** 10.1111/irv.13225

**Published:** 2024-02-05

**Authors:** Luke W. Meredith, Mustafa Aboualy, Rachel Ochola, Mehmet Ozel, Abdinasir Abubakar, Amal Barakat

**Affiliations:** ^1^ Infectious Hazard Management, Department of Health Emergency World Health Organization, Eastern Mediterranean Regional Office Cairo Egypt

**Keywords:** capacity building, COVID‐19, Eastern Mediterranean Region, laboratories, public health, SARS‐CoV‐2

## Abstract

The Eastern Mediterranean Region (EMR) faces ongoing challenges in its public health system due to limited resources, logistical issues, and political disruptions. The COVID‐19 pandemic accelerated the need for stronger laboratory capacities to handle the increased demand for testing. In a phased response, EMR countries utilized the National Influenza Centers to rapidly establish and scale molecular testing for SARS‐CoV‐2, the causative agent of COVID‐19. The expansion of capacity included strong collaborations between public health bodies and private and academic sectors to decentralize and expand testing to the subnational level. To ensure that the quality of testing was not impacted by rapid expansion, national and subnational laboratories were enrolled in external quality assurance programs for the duration of the response. Implementation of genomic surveillance was prioritized for variant tracking, leading to the establishment of regional sequencing reference laboratories and the distribution of MinION sequencing platforms to complex emergency countries who previously had limited experience with pathogen sequencing. Challenges included a lack of technical expertise, including in implementing novel diagnostic assays and sequencing, a lack of bioinformatics expertise in the region, and significant logistical and procurement challenges. The collaborative approach, coordinated through the WHO Eastern Mediterranean Regional Office, enabled all 22 countries to achieve SARS‐CoV‐2 diagnostic capabilities, highlighting the pivotal role of laboratories in global health security.

## 1 BACKGROUND

The Eastern Mediterranean Region (EMR), encompassing 21 member states and one territory, has been a pivotal area for public health efforts within the World Health Organization (WHO) Eastern Mediterranean Regional Office (EMRO) in the past two decades. The region has a diverse population of 730 million people and faces intricate challenges in maintaining effective public health and laboratory networks.^1^ These challenges range from constraints on financial and technical resources to challenging logistics, political commitments, and a lack of quality management systems. These factors present a complex landscape where laboratory diagnostics and response to health crises are concerned. Amidst these challenges, EMR laboratory networks bear the responsibility of detecting and reporting public health events under the International Health Regulations (IHR:2005), emphasizing the critical need for functional laboratory capacities.^2^ Indeed, while the COVID‐19 pandemic presented the greatest global threat over the past 3 years, over 60 other outbreaks of high‐threat pathogens have been reported in the region,^3^ including bacterial diseases such as cholera which impacted over 50% of the region in the past 2 years and other viral outbreaks such as Crimean Congo Hemorrhagic Fever (CCHF) which is currently circulating in multiple countries,^3^ emphasizing the burden that laboratory networks faced during the pandemic.

## 2 PANDEMIC EMERGENCE AND EMR RESPONSE

The advent of the COVID‐19 pandemic in late 2019^4^ served as a crucible for the EMR's public health resilience. The pandemic's impact was swift and global, necessitating rapid adaptation and expansion of diagnostic capacity both globally and within the region. In January 2020, the United Arab Emirates reported the first COVID‐19 cases within the region,^5^ swiftly followed by other countries. By April 2020, the entire EMR landscape was grappling with COVID‐19 infections. A marked disparity between global and EMR case counts emerged, with the latter contributing 3.07% of global cases with a case fatality rate (CFR) of 1.5%, compared with a global CFR of closer to 0.5%,^6^ with the caveat that the global and regional CFRs are likely to be estimates due to challenges with data collection globally. This emphasized the vulnerability of the regional population to the pandemic, with challenges facing all aspects of public health. The WHO EMRO, in collaboration, mounted a series of responses outlined in a separate publication, but a key aspect of response was a phased enhancement of regional laboratory capacity.

## 3 WHO/EMRO PHASED RESPONSE TO LABORATORY CAPACITY

**FIGURE 1:**
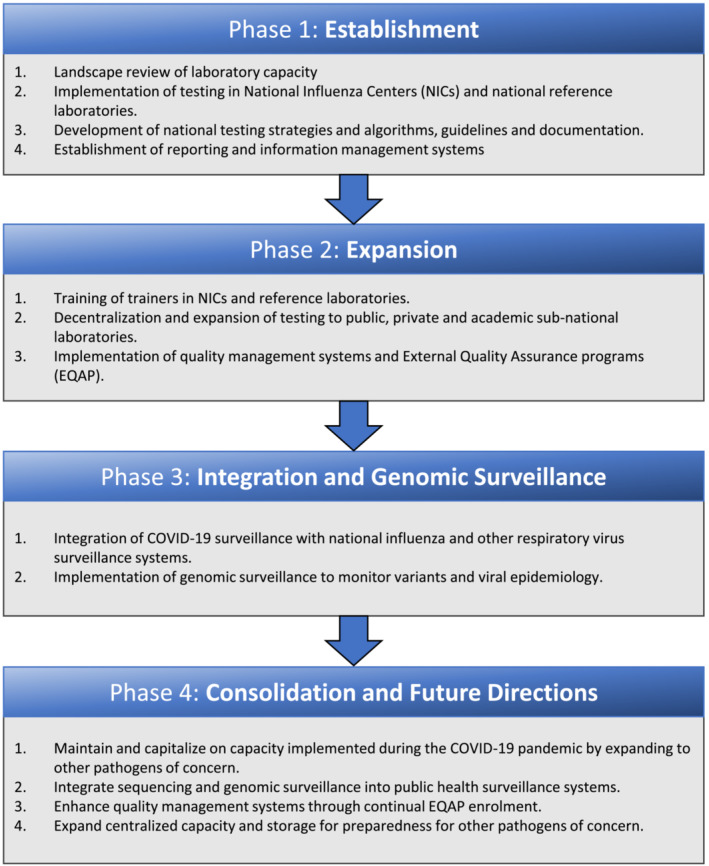
Phases of laboratory enhancement in the Eastern Mediterranean Region (EMR) countries during the COVID‐19 pandemic. The WHO EMRO supported a phased rollout of laboratory testing, including expansion of national and regional laboratory capacity, integration of public and private health laboratories, and planning for future sustainability of capacity.

Laboratory detection is a key aspect of the public health responses. It underpins clinical, surveillance, and epidemiology efforts during disease responses. The WHO/EMRO Infectious Hazard Preparedness division (IHP), supported by global stakeholders including World Bank, US Agency for International Development (USAID), US Centers for Disease Control and Prevention (US CDC), UK Health Services Agency (UKHSA), and Japan Center for Disease Control (Japan CDC), focused attention on bolstering laboratory capacity across the region, to enable rapid and reliable diagnostics to inform the unfolding public health actions. The response was driven through three phases (Figure 1): assessment and establishment of testing capacity, expansion of testing facilities at the subnational level, then implementation of genomic surveillance.

1. Assessment and establishment of testing capacity at the national level.

**FIGURE 2:**
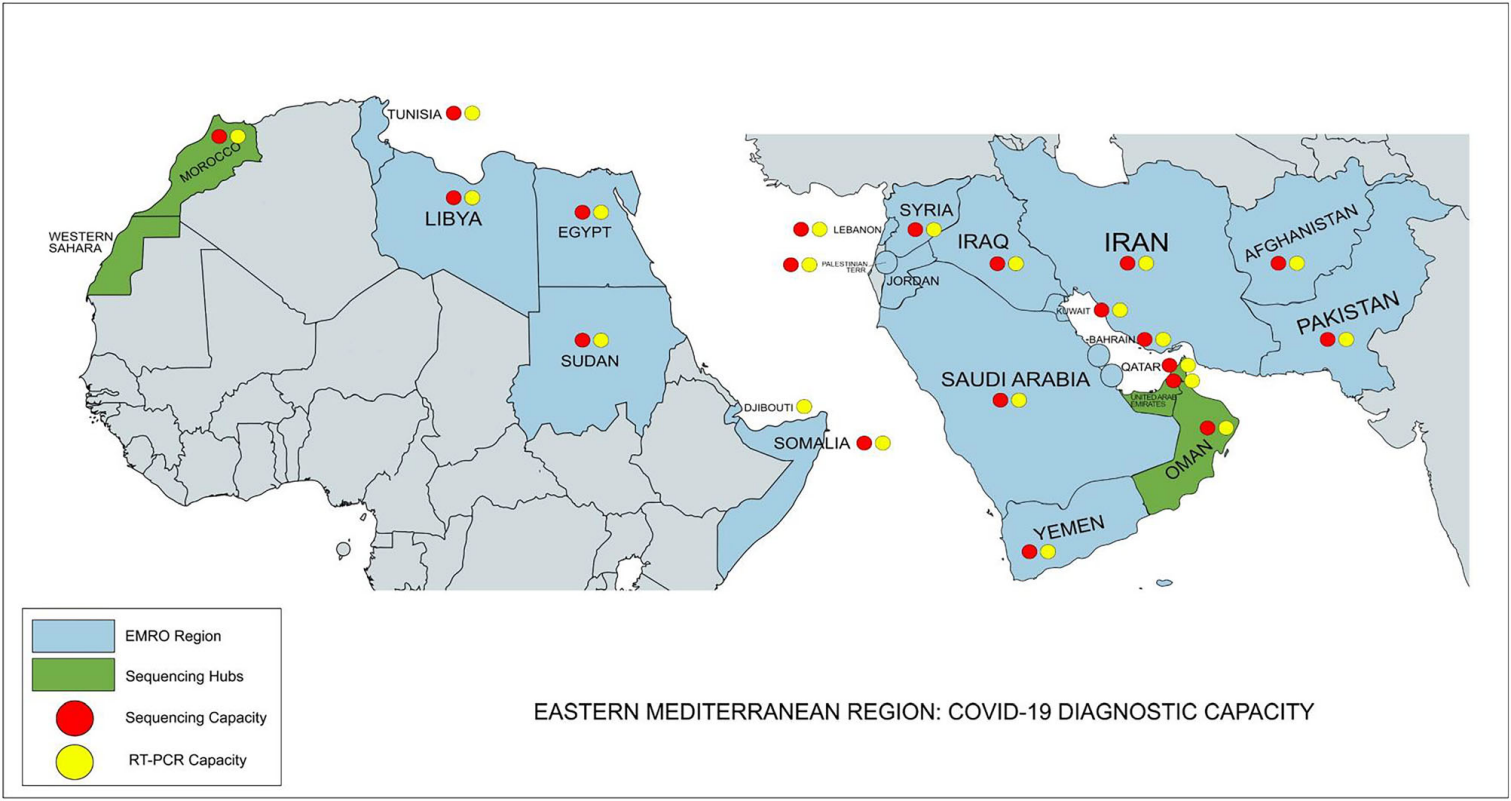
Status of SARS‐CoV‐2 laboratory capacity in the Eastern Mediterranean Region (EMR). All 22 member states across EMR now have laboratory capacity for molecular detection of SARS‐CoV‐2, while 21/22 countries have capacity for sequencing.

Prior to the advent of rapid tests,^7^ molecular testing through polymerase chain reaction (PCR) was the primary mode of detection, requiring a functional molecular laboratory operating following good laboratory practice to ensure the accuracy of the results provided. A landscape review at the start of the pandemic showed that only 18% of EMR member states had capacity for SARS‐CoV‐2 detection, either through academic or public health infrastructure.^8^ However, 19/21 countries had designated the National Influenza Centers (NIC) which had the capacity for high‐throughput and high‐quality molecular diagnostics (Figure 2). NICs are WHO‐recognized institutions that are responsible for laboratory surveillance of influenza, influenza‐like illnesses (ILI), and severe acute respiratory illnesses (SARI).^9^ The teams operating in these centers had extensive experience handling respiratory pathogens and were ideally placed to lead national efforts for COVID‐19 diagnostics.

Working with the Influenza Division of the US CDC, under the remit of the Pandemic Influenza Preparedness (PIP) Framework,^10^ capacity and expertise in the NICs were redirected toward COVID‐19 detection and diagnosis. Priority was given to ensuring that national experts were able to competently perform SARS‐CoV‐2 diagnostic PCR assays, as well as having sufficient expertise to share their knowledge with subnational facilities through a “train‐the‐trainer” approach. This approach was taken to ensure that national laboratories could support the expansion of testing to subnational laboratories in the event of a surge in cases.

The WHO EMRO then shifted focus to the logistical task of supplying and equipping laboratories with sufficient tests to confirm cases as they emerged. Working in collaboration with the School of Public Health at the University of Hong Kong, the WHO EMRO mobilized an emergency stock of diagnostic kits that were sufficient for detection of emergent cases in countries across the region. The kits, consisting of lyophilized primers, probes and PCR reagent, were developed and donated by the University and were supplied lyophilized to allow ease of distribution. This was a critical consideration as cold‐chain transport is severely limited in conflict or emergency countries, of which 50% of countries in the EMR division are categorized.

By 20 February 2020, 6 weeks after the PHEIC was declared, NICs in country had received kits and were able to capitalize on their experience, existing infrastructure and the emergency training and support to commence testing by February 22. The remaining two countries, who did not have existing NICs, instead relied on international referrals, while developing their internal capacity to use the assays.

While the kits supplied by the University were a useful stopgap measure for emergency use, the WHO continued to work at a global level to mobilize commercial molecular assays. These have the benefit of more extensive quality control, with assays needing to achieve CE or FDA markings in order to be purchased or distributed by the WHO.^11^ The WHO selected the TIB Molbiol Assay,^12^ based on the research of Drosten et al.,^13^ and supported the global distribution of the assays to all affected countries by the end of February 2020.

This expansion of commercially available assays was particularly important as it reduced the dependency on donations from academic institutions and improved the availability of assays for countries actively under sanction or embargo, who have the constant threat of stock depleting before new assays can be procured. This phased approach of utilizing academic or research‐based assays as the first line of testing proved to be highly successful during the outbreak, and similar models could be employed in future outbreaks.

2 Expansion of testing facilities and quality assurance at the subnational level.

As the scale of the pandemic became apparent, decentralized testing became critical to national and international surveillance and response efforts. To address this, with support from national and international donors, EMR countries operationalized new laboratories, including modular and mobile testing units, particularly to support remote areas. Over the course of the pandemic, the number of labs expanded from 22 national laboratories, to over 300 laboratories with molecular testing capacity (Aboualy et al., accepted for publication in this supplement). This expansion was underpinned by the engagement of private, research, and academic laboratories who had significant technical expertise to offer to complement public health efforts. This unprecedented expansion of capacity presented significant logistical challenges and also required careful monitoring and evaluation to ensure that the quality of the results reported was maintained.

To facilitate this, an External Quality Assurance Programme (EQAP) was instrumental in maintaining testing quality, with reference laboratories contributing to refining processes and supporting ongoing improvement in sub‐national laboratories. A total of 309 laboratories participated in the EQAP across the region, with 95.5% and 92.1% of laboratories achieving 100% concordant results in the first and second EQAP rounds, respectively (Aboualy et al., accepted for publication in this supplement). Moreover, the laboratory capacity component of the International Health Regulations (IHR) also saw substantial improvement, increasing from 69% in 2019 to 84% in 2021, highlighting that while many new laboratories were established, there was still a strong focus on ensuring the quality in the testing.

The incorporation of private and academic laboratories was a key aspect of the success of the response in the region. Public/private collaborations, such as that observed in Jordan between Biolabs and the Ministry of Health (Abu Dayyeh et al., accepted for publication in this supplement), and the academic excellence of laboratories in countries such as Qatar and Oman allowed for a significantly quicker scaling of testing capacity than establishing entirely new laboratories through public health laboratory programs.

There were significant challenges, including the implementation of the EQA program, and the logistical challenges of supplying such a diverse network of laboratories, as well as the challenge of incorporating data into national reporting networks. However, the efforts taken to address these challenges, such as the implementation of novel IT platforms for data integration and reporting (such as observed in the manuscript from Abu Dayyeh et al., in this supplement), and streamlined logistics and cold chain for dissemination of assays, mean that the region has overall improved capacity for response to outbreaks in future.

3 Implementation of genomic surveillance for variant monitoring and assessment.

The pandemic highlighted the challenges of viral evolution, with the virus genome changing rapidly due to factors such as vaccine and therapeutic response and widespread dissemination, all of which put pressure on the virus to evolve.^14^ Over the course of the pandemic, the benefit of sequencing and genomic surveillance became clear, particularly for monitoring variants of concern (VOCs)^15^ and vaccine responses, as well as tracking the spread of variants globally.^16^ The EMR, recognizing the necessity of monitoring variants, embarked on the third phase of laboratory capacity enhancement which involved the implementation of sequencing and genomic surveillance throughout the region.

During the regional laboratory landscape assessment, it was found that 13 out of the 22 countries in the region had sequencing capacity, either in national public health laboratories, academia, or private industry. However, many of these countries lacked experience in the rollout of pathogen‐specific sequencing assays or platforms or faced challenges with data analysis and bioinformatics when the sequencing was successful. The WHO EMRO therefore supported the establishment of three regional sequencing reference laboratories, located in Morocco (Laboratoire de Virologie at the Institut National d'Hygiene, Rabat), Oman (Central Public Health Laboratory, Muscat), and the United Arab Emirates) Sheikh Khalifa Medical City (SKMC) Reference Laboratory for Infectious Diseases, Abu Dhabi). These laboratories had the expertise, experience, and technical capacity to implement sequencing on multiple platforms, including Illumina®, Ion Torrent®, and Oxford Nanopore Technology and to provide the bioinformatics support needed to produce meaningful public health data. These facilities also had close logistical connections with other countries in the region, allowing for transfer of samples as needed to provide regional support for sequencing.

Throughout the pandemic, these reference centers operated as training platforms for countries in the region. To expand sequencing capacity further, the WHO EMRO provided the Oxford Nanopore MinION platform to complex emergency countries, who lacked the capacity to monitor or track variants that emerged nationally. The MinION was selected specifically for the compact size, robust workflows, and ease of distribution, which allows it to be used both in large national laboratories and smaller subnational facilities who may have limited power infrastructure. Logistical support was provided to ensure that the equipment and reagents were delivered using appropriate cold‐chain delivery systems, while IT and bioinformatic support were also prioritized to ensure that the data generated could be added to national and international efforts to monitor the virus. Two regional training courses were then held in Oman and UAE specifically to support sequencing of SARS‐CoV‐2, presented collaboratively by WHO and the UK New Variant Assessment Platform (NVAP), a very experienced bioinformatics support team from the UK Health and Security Agency (UKHSA). During these courses, all countries in the region with either established or new sequencing capacity from Oxford Nanopore were given comprehensive training in the laboratory protocols associated with sequencing, as well as hands‐on training with the bioinformatics pipelines needed to assemble viral genomic data. By the end of 2022, 21 out of the 22 countries in the region were actively contributing data to the Global Initiative on Sharing All Influenza Data (GISAID) EpiCoV platform, demonstrating the success of this rollout across the region.

While the implementation of COVID‐19 sequencing was largely successful, the largest single challenge to widespread genomic surveillance remains the challenge of bioinformatics and data analysis. Centralized platforms such as GISAID provide an excellent resource to deposit data and to get an overview of the international response but can be challenging to assess for local or regional data assessment. It should also be noted that while producing genomes can be automated or streamlined into simple processes, the production of meaningful phylogenetic analyses remains challenging, as does the translation of such data into useful public health information. The WHO, both in the EMRO region and internationally, are aware of these challenges and are looking to address them through the implementation of networks such as the International Pathogen Surveillance Network (IPSN) or by implementation of the global strategy for genomic surveillance,^17^ or indeed the WHO EMRO regional genomic strategy (Meredith et al., manuscript accepted for publication in this supplement), with the goal of ensuring that the region is prepared to respond to the next outbreak when it occurs.

## 4 FURTHER CHALLENGES AND REFLECTIONS

The EMR's journey toward strengthening laboratory capacity yielded tangible results. The collaborative and systematic approach led to all 22 countries achieving SARS‐CoV‐2 diagnostic capabilities using molecular diagnostics, as well as 21 out of the 22 countries having genomics capacity to contribute to international efforts to further understand the evolution of SARS‐CoV‐2. With the establishment of subnational testing networks and the integration of laboratories into national and regional networks, responsiveness was significantly enhanced, with turnaround times reduced from days or weeks in some cases to hours. The EQAP results underscored the maintenance of quality across the expanded laboratory network, in spite of the rapid expansion.

While this was a significant success for the region, it did not come without significant challenges. National public health agencies, particularly those currently in conflict or embargoed by international communities, continually face severe restrictions in acquiring reagents or equipment. This is a common issue in the region and is constantly under review by organizations such as WHO EMRO to ensure that these delays can be reduced to improve the response to outbreaks of infectious disease in future. These commercial impediments due to political challenges and conflict present one of the biggest threats to pandemic preparedness and need continual review to protect the public as a whole. The WHO EMRO and other international stakeholders are working together to ensure that the WHO Dubai Logistics Hub has the capacity to respond to threats as they emerge and to support the region while national procurement pipelines are implemented for high‐threat pathogens, particularly for complex emergency countries.

Furthermore, there is a continual and ongoing issue with the implementation of sample management systems. While decentralization of testing allows for rapid expansion of testing capacity, the collection and transfer of the metadata associated with samples, such as critical patient details, date of disease onset, and other parameters, are often neglected. This means that data can be obtained about positivity but provide less useful information for surveillance efforts, particularly when sequencing is being used. The primary purpose of sequencing is surveillance, but it needs to be continually emphasized that without a functional data collection system, ideally digital to ensure rapid dissemination of data, then the utility will be limited. The WHO EMRO and other stakeholders will continue to work with the region to implement digital laboratory information systems, such as the EMFLU or DHIS2 platforms,^18^ which will allow the region to more rapidly respond to threats as they emerge.

The lack of expertise in bioinformatics and data analysis for genomic data also presents a key challenge. Training courses are ongoing to fill this gap across the region, initially through the three hub locations, but with the goal of expanding regionally in the near term. Genomic surveillance is a powerful tool that will be one of the frontline responses for rapid outbreak detection and confirmation and for future pandemic responses, and ongoing efforts need to be maintained to again ensure that the region can respond to emerging threats in near real‐time. The data provided by genomic surveillance are key to developing vaccines and diagnostic tools, two of the frontline defenses for pandemic response, and the WHO/EMRO will continue to work with MS to ensure that this capacity is maintained during the wind‐down of the COVID‐19 pandemic response.

## 5 CONCLUSION

The EMR's response to the COVID‐19 pandemic exemplifies the region's dedication to enhancing laboratory capacity for public health preparedness. Through collaboration, investment, and systematic expansion, all 22 countries achieved SARS‐CoV‐2 diagnostic capabilities, underpinning timely and reliable data for public health actions. The multifaceted approach, encompassing national, regional, and international collaboration, serves as a blueprint for effectively addressing emerging health challenges. As the region endeavors to integrate enhanced laboratory capacities into routine surveillance, the groundwork has been laid for an enduring legacy of resilience against future epidemics and emerging pathogens. The lessons learned from this pandemic underscore the importance of adaptive strategies and investments in laboratory networks as cornerstones of global health security.

## AUTHOR CONTRIBUTIONS

A. A., A. B., R. O., and M. O. conceptualized the paper. L. M. drafted the manuscript with assistance from M. A., R. O., and M. O. P. O provided editing and proofing support. All authors reviewed and contributed to subsequent drafts for important intellectual content and approved the final manuscript. The authors alone are responsible for the views expressed in this publication, and they do not necessarily represent the decisions or policies of WHO/EMRO.

## CONFLICT OF INTEREST STATEMENT

All authors are employed by WHO/EMRO.

## Data Availability

Data sharing is not applicable to this article as no new data were created or analyzed in this study.
